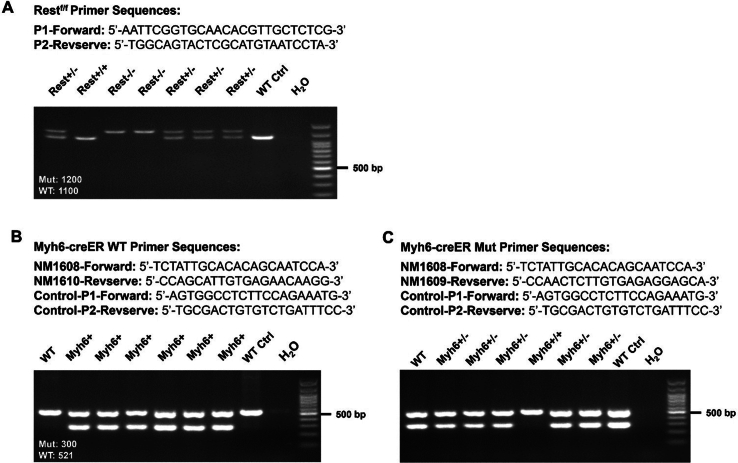# The silencing transcription factor REST targets UCHL1 to regulate inflammatory response and fibrosis during cardiac hypertrophy

**DOI:** 10.1016/j.gendis.2023.101183

**Published:** 2023-11-25

**Authors:** Wenze Cao, Huan Liu, Ye Xu, Sangyu Hu, Yujie Yang, Li Li, Luying Peng

**Affiliations:** a State Key Laboratory of Cardiology, Shanghai East Hospital, Tongji University School of Medicine, Shanghai 200120, China; b Institute of Medical Genetics, Tongji University, Shanghai 200092, China; c Heart Health Center, Shanghai East Hospital, Tongji University School of Medicine, Shanghai 200120, China; d Department of Medical Genetics, Tongji University School of Medicine, Shanghai 200092, China; e Research Units of Origin and Regulation of Heart Rhythm, Chinese Academy of Medical Sciences, Beijing 100730, China

RE1 silencing transcription factor (REST) plays a key role in embryonic development and fetal cardiac gene reactivation.^1^ However, understanding of the role of REST in cardiac remodeling is very limited. A recent study has shown that cardiac-specific REST knockout increases Gαo expression, and impairs Ca^2+^ processing in ventricular myocytes, leading to cardiac dysfunction.^2^ Moreover, REST could bind to the neuron-restrictive silencer element region of the UCHL1 (ubiquitin carboxy-terminal hydrolase L1) gene promoter and regulate the expression of UCHL1.^3^ A recent study has reported that UCHL1 is significantly up-regulated in hypertrophic hearts, and positively regulates cardiac hypertrophy through stabilizing epidermal growth factor receptors.^4^ Here, we found that cardiac-specific REST knockout (REST cKO) mice showed more severe fibrosis and inflammation following pressure overload conditions. REST deficiency up-regulated UCHL1 expression, which then exacerbated cardiac hypertrophy. Whatmore, the application of UCHL1 inhibitor LDN-57444 in the REST cKO mice could alleviate fibrosis and inflammation in hypertrophic hearts via the NF-κB (nuclear factor-κB) and STAT3 (signal transducer and activator of transcription 3) signaling pathways.

In the present work, we first detected the expression status of REST in both hypertrophic heart and cardiomyocytes. The mouse model induced by intraperitoneal injection of Ang II showed significant cardiac hypertrophy (Fig. S1A, B) and dysfunction (Fig. S1C, D), but the mRNA and protein levels of REST were found to be significantly down-regulated in the hypertrophic heart tissue (Fig. S1E, F). Similarly, the transverse aortic constriction (TAC) model with cardiac hypertrophy (Fig. S1G, H) and heart dysfunction (Fig. 1A; Fig. S1I) also exhibited an obvious decrease in REST expression level (Fig. 1B, C). Furthermore, the challenge of primary neonatal rat cardiomyocytes (NRCMs) with Ang II for 48 h augmented cardiomyocyte hypertrophy (Fig. S1J), resulting in a consistent expression pattern for both hypertrophic markers (Fig. S1K) and REST (Fig. S1L, M) with that observed in TAC heart. Overall, these *in vivo* and *in vitro* data suggested that REST might play a potential role in the regulation of cardiac hypertrophy.

**Figure 1:**
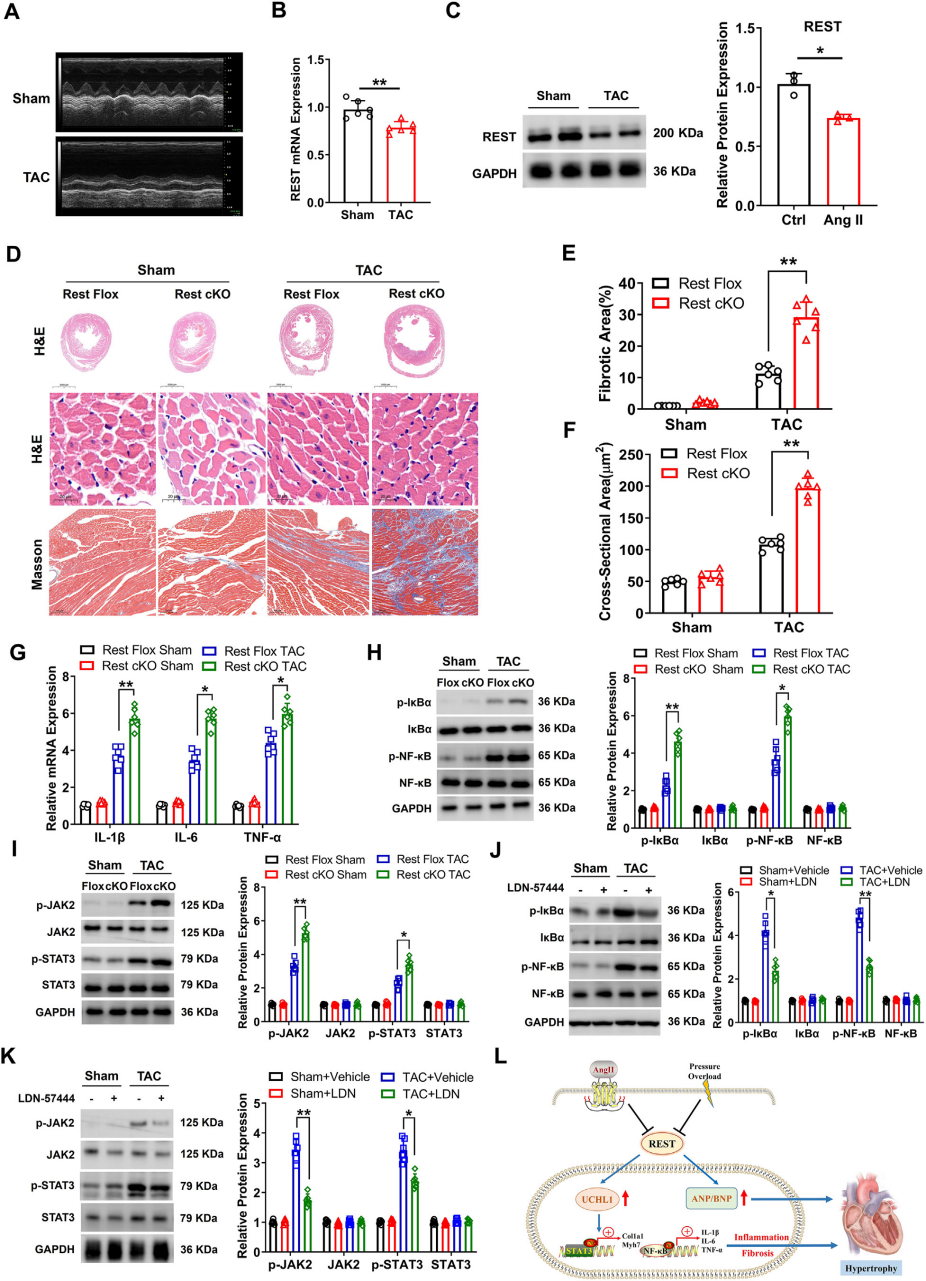
REST regulates inflammatory response and fibrosis in cardiac hypertrophy via targeting UCHL1. **(A)** Representative M-mode echocardiography of left ventricular chamber (*n* = 6). **(B, C)** qRT-PCR and Western blot results of REST expression at week 4 after TAC surgery (*n* = 6). **(D)** The tissue status in the four-week TAC hearts of REST Flox and REST cKO mice by hematoxylin and eosin staining and Masson's trichrome staining (*n* = 6). Scale bar: 2000 μm for the top sections, 20 μm for the mid sections, and 100 μm for the bottom sections. **(E, F)** Quantitative results of average cross-sectional areas and collagen accumulation from the indicated groups. **(G)** Quantitative PCR results of inflammatory factors (IL-1β, IL-6, and TNF-α) in REST Flox and REST cKO mice (*n* = 6). **(H)** The phosphorylation levels of IκBα and NF-κB in REST Flox and REST cKO TAC mice of four weeks (*n* = 6). **(I)** The phosphorylation levels of JAK2 and STAT3 in REST Flox and REST cKO TAC mice of four weeks (*n* = 6). **(J, K)** The phosphorylation levels of IκBα and NF-κB or JAK2 and STAT3 in REST cKO TAC mice with LDN-57444 administration (*n* = 6). **(L)** The schematic working model of REST regulating inflammatory response and fibrosis in cardiac hypertrophy. All data represent mean ± standard deviation. ∗*p* < 0.05, ∗∗*p* < 0.01.

To evaluate the effect of REST on hypertrophy *in vitro*, NRCMs were infected with an adenovirus vector expressing small interfering RNA against REST (Fig. S2A). Notably, inhibition of REST further enhanced the Ang II-induced expression of hypertrophic markers, including atrial natriuretic peptide (ANP) and B-type natriuretic peptide (BNP) (Fig. S2B). In contrast, forced expression of REST with pCMV-Flag-REST (Fig. S2C) in NRCMs markedly repressed the Ang II-induced transcriptional levels of ANP and BNP, compared with those in the control group (Fig. S2D). Taken together, RSET shows a protective role in cardiomyocytes against Ang II-induced hypertrophy.

Given the *in vitro* results, we next evaluated the physiological consequences of REST deletion *in vivo*. We generated cardiac-specific REST conditional knockout mice (REST f/f: α-MHC-CreER), which all showed a healthy condition. After being injected with tamoxifen for five consecutive days, REST cKO mice and Flox ones were subjected to TAC for four weeks to generate a hypertrophic model (Fig. S3A). Hematoxylin and eosin staining and Masson's trichrome staining showed that REST cKO mice developed obvious cardiomyocyte hypertrophy with collagen accumulation compared with the Flox group (Fig. 1D–F). Moreover, hypertrophic parameters, including the ratios of heart weight to body weight, levels of ANP and BNP, left ventricular ejection fraction and fractional shortening, and levels of collagen I and collagen III were all further aggravated in REST cKO mice with TAC (Fig. S3B–G). Notably, in the first four weeks, tamoxifen-induced REST cKO mice did not show any significant difference in mortality and echocardiography (Fig. S3F–H), and a few deaths only occurred five weeks later. However, TAC could induce an increase in mortality in REST cKO mice (Fig. S3H). These results indicated that REST deletion really exacerbated cardiac hypertrophy and fibrosis.

REST has been proven to exert a role in regulating inflammatory responses in the nervous system,^5^ we hence try to understand whether REST mediates inflammation pathways involved in cardiac hypertrophy. We found that the deletion of REST promoted up-regulation of inflammatory factors interleukin (IL)-1β, IL-6, and TNF (tumor necrosis factor)-α induced by TAC (Fig. 1G). Similar results were also observed in *in vitro* models upon inhibiting REST in Ang II-challenged NRCMs (Fig. S4A) and could be obviously reversed by forced expression of REST (Fig. S4B). Next, we try to clarify whether REST mediated the change of inflammatory factors involved in NF-κB signaling, a classical pathway of the inflammation process. The phosphorylation level of IκBα and NF-κB (p65) was found to have an increase in TAC mice in the absence of REST (Fig. 1H), which was also verified in Ang II-induced cardiomyocytes (Fig. S4C). Moreover, the STAT3 pathway, which can regulate inflammation and fibrosis in cardiac hypertrophy, and JAK2 were further activated in the absence of REST in TAC mice (Fig. 1I). Overexpressing REST could conversely inhibit the phosphorylation of STAT3 and JAK2 (Fig. S4D). Hereby, REST alleviates inflammation response in cardiac hypertrophy via triggering NF-κB and JAK/STAT pathways.

REST generally interacts with the promoter of the UCHL1 gene to modulate its expression.^3^ We found here that the absence of REST induced an up-regulation of UCHL1 (Fig. S5A). To further evaluate UCHL1's role in cardiomyocyte hypertrophy, LDN-57444, a specific small-molecule inhibitor of UCHL1, was used to challenge TAC mice. All REST cKO mice were subjected to TAC or Sham for one week and then randomized to be administered vehicle only or LDN-57444 for an additional three weeks (Fig. S5B). Consistently, REST deletion aggravated cardiac dysfunction and exacerbated myocardial inflammation, whereas these pathological changes were remarkably reduced when treated with LDN-57444 (Fig. S5C–G). In addition, the administration of LDN-57444 attenuated phosphorylation levels of NF-κB and STAT3 in REST cKO-TAC models (Fig. 1J, K). Taken together, these results indicate that REST mediates UCHL1 to be involved in the cardiac remolding process.

In conclusion, this study confirms the novel effect of REST in preventing heart remodeling via mediating NF-κB and STAT3 pathways (Fig. 1L).

## Ethics declaration

The animal studies in this article were approved by the Animal Ethical and Welfare Committee of Tongji University.

## Author contributions

L.P. and L.L. conceived and designed the project; W.C. performed major experiments. Huan Liu carried out part of the experiments; Ye Xu and Sangyu Hu analyzed the data; All the authors were involved in the discussion of results. Wenze Cao wrote the manuscript. Luying Peng and Yujie Yang revised the manuscript. All authors read and approved the manuscript.

## Conflict of interests

The authors have declared that no conflict of interests exists.

## Funding

This work was supported by grants from the 10.13039/501100001809National Natural Science Foundation of China (No. 32071109, 82070270, M−0048) and the Shanghai Committee of Science and Technology (China) (No. 21ZR1467000, 22ZR1463800).

## Figures and Tables

**figs1:**
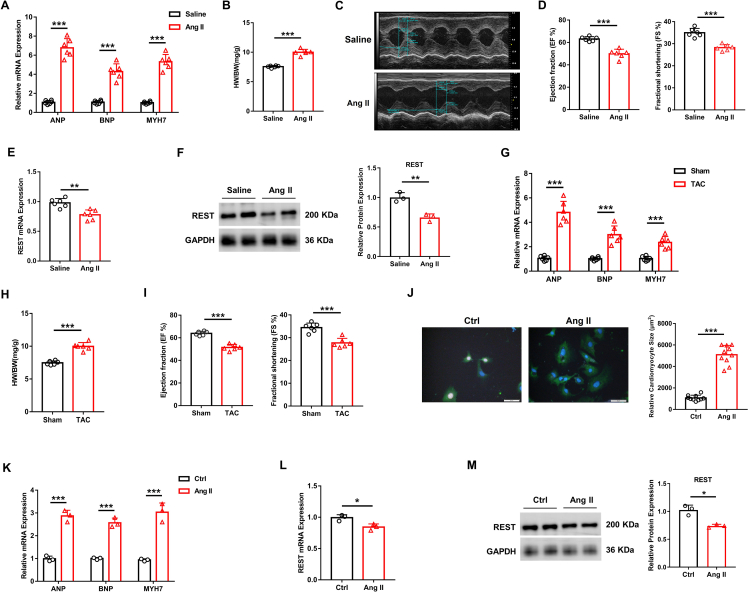


**figs2:**
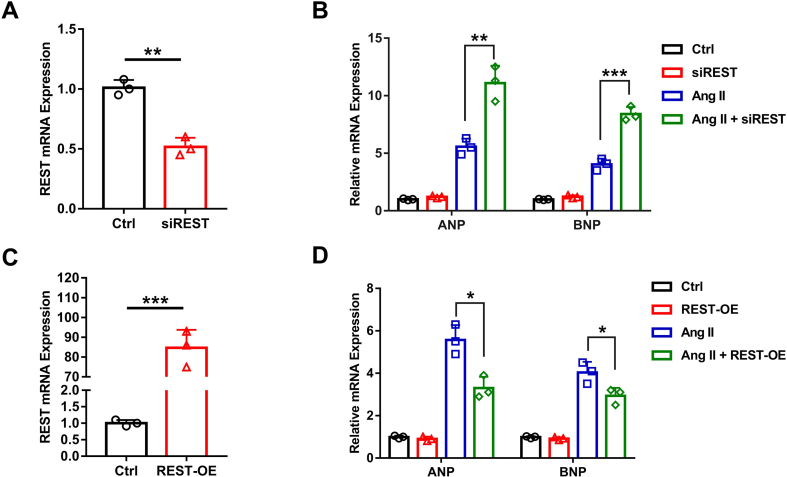


**figs3:**
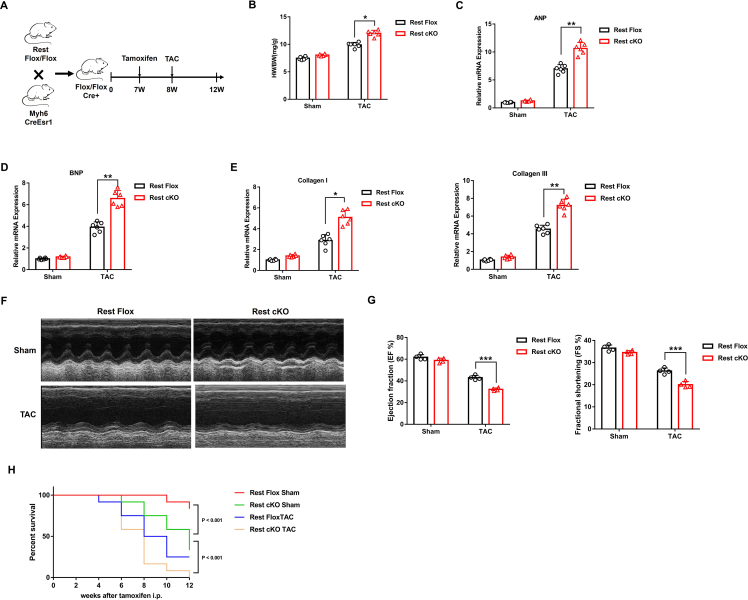


**figs4:**
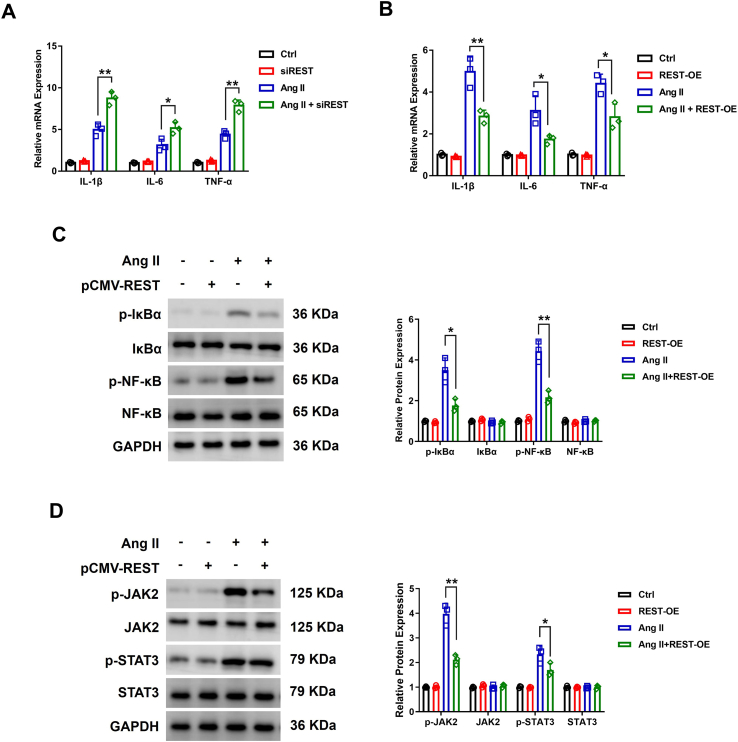


**figs5:**
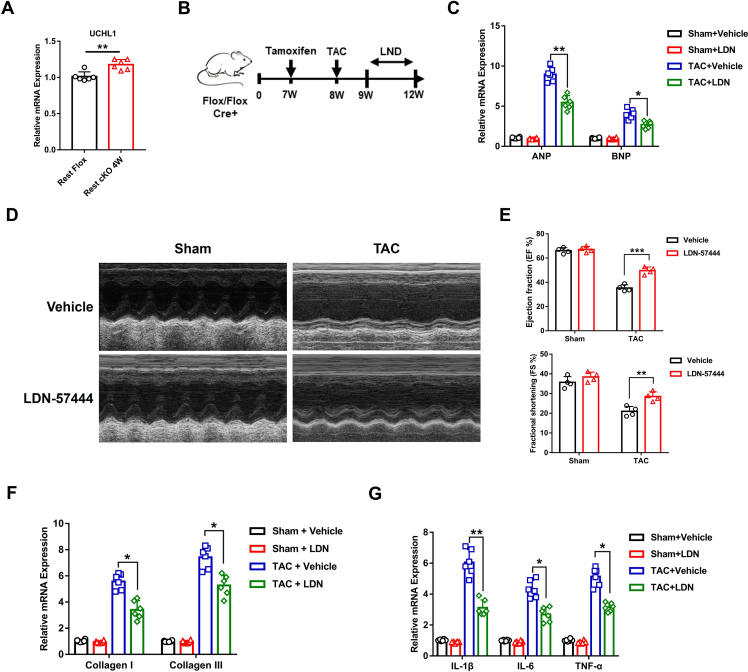


**figs6:**