# Supplementary material for: The silencing transcription factor REST targets UCHL1 to regulate inflammatory response and fibrosis during cardiac hypertrophy
Source: Genes Dis. 2023 Nov 25;11(6):101183. doi: 10.1016/j.gendis.2023.101183 (PMC11327510; doi:10.1016/j.gendis.2023.101183)
Supplement: Multimedia component 1 [file mmc1.docx]

**Supplementary Materials and Methods**

**Animals**

Cardiac-specific REST conditional knockout mice (REST f/f: α-MHC-Cre-ER) were obtained from Shanghai Model Organisms Center, INC. Genotyping method were done as supplementary data (Figure S6). The 6-8 week old C57BL/6 male mice were injected intraperitoneally with Ang II (15 mg/kg·d) for 21 days for preparing hypertrophic model. To generate tamoxifen-inducible cardiac-specific REST-KO mice, tamoxifen was administered (75mg/kg) via intraperitoneal injection once every 24 hours for a total of 5 consecutive days. Then the mice were subjected to sham or TAC operation. After 2 weeks, UCHL1 inhibitor LDN-57444 (40 μg/kg, one time per day) was intraperitoneally injected into mice for additional 3 weeks. All animal experiments and procedures were performed in accordance with the National Institutes of Health guide for the care and use of laboratory animals and approved by Institutional Animal Care and Use Committee at Tongji University (Approval No: TJAA00220402). Mice were euthanized by cervical dislocation for cardiac tissue collection.

**Preparation of hypertrophic cardiomyocytes**

Neonatal Sprague Dawley rats were purchased from Slaccas Company (Shanghai, China). Primary neonatal rat cardiomyocytes (NRCMs) were isolated according to previous description [1]. Briefly, the ventricles were separated and cut into pieces, then cells were digested with 0.1% type II collagenase (Invitrogen) and 0.1% trypsin (Gibco) and gently shaken at 37°C. the supernatant was resuspended with DMEM including 10% FBS (Gibco) and cultured at 37°C with 5% CO_2_ for 1 hour. Non‐adherent cells (myocytes) were collected and cultured in fresh medium (Gibco) harbouring 0.1 mM BrdU (sigma) for 48 hours. To induce hypertrophy, NRCMs were treated with 1µM Ang II (Sigma, Saint Louis, MO, USA) for 48 h.

**Cell transfection**

NRCMs were seeded at 2 × 10^6^ in 6-well plates, and then cultured with serum for 24h, followed by starving with serum‐free DMEM overnight. For gene silencing studies, siRNAs (Genepharma) were transfected into NRCMs at a final concentration of 50 nM using Lipofectamine 3000 (Invitrogen, Carlsbad, CA, USA). 4-6 hours later, cells were cultured with fresh medium for another 24 hours before next step experiments. For overexpression, pCMV-Flag and pCMV-Flag-REST (GENECHEM, Shanghai, China) was transfected respectively into NRCMs using Lipofectamine 3000. The follow-up experiments were conducted in the same way as described earlier.

**Transverse aortic constriction surgery and echocardiography**

REST Flox and REST cKO mice were divided into two groups: the sham group and TAC group. The brief operation process is described as follows: first of all, the surgical tools were autoclaved, and then mice fur were shaved from the neck to the mid-chest after anesthesia with isoflurane, while mice were connected to a respirator cycling at 120–140 breaths/min. After fully exposing the surgical field, the 27 G syringe needle was placed parallel to the transverse aorta and knotted surround tightly. Finally, the chest and skin were closed in sequence by suture. The entire procedure in the sham group was the same except for the ligation of the aorta. All operated mice were fed in the same condition. After 4 weeks, echocardiography was performed with Vevo 770 ultrasound system (Visual Sonics Inc.) and with M-mode to detect the cardiac function. All of mice were performed under anaesthesia (1.5%‐2% isoflurane, 2 L/min oxygen flow rate).

**Quantitative Real-Time PCR**

Total RNA was extracted from tissues or NRCMs with TRIzol reagent (Invitrogen), and cDNA was synthesized using PrimeScript RT reagent Kit with gDNA Eraser (TaKaRa). Expression levels of genes were determined using TB green premix Ex Taq (TaKaRa) on the Bio‐Rad CFX connect™ real‐time PCR system with specific primers. Primers are synthesized by Sangon Biotech, and all of the sequences of the qPCR primers were shown in table S1.

**Western blot**

The total protein was extracted with RIPA lysis buffer and concentration of protein was measured using the BCA Protein Assay kit (TaKaRa). The protein samples were resolved by SDS–polyacrylamide gel electrophoresis (SDS-PAGE) gels and transferred to 0.45um PVDF membranes (Millipore, USA). Then, the membranes were blocked in 5% BSA and incubated at 4 °C overnight with primary antibodies. After washed, the membranes were incubated with the appropriate secondary antibodies for 1h. The signals of protein were imaged with Amersham Imager 600. The primary and secondary antibodies used in this study are shown as follows and in table S2.

**Statistical analysis**

All data were analyzed with Prism 7.0 (GraphPad Software Inc). Differences between groups were evaluated for statistical significance using Student’s t test was used to compare the differences of two groups. P values less than 0.05 was considered statistically significant.

**References**

[1] Li, Z., H. Zhu, C. Liu, et al., GSK-3β inhibition protects the rat heart from the lipopolysaccharide-induced inflammation injury via suppressing FOXO3A activity. J Cell Mol Med, 2019. 23(11): p. 7796-7809.

**
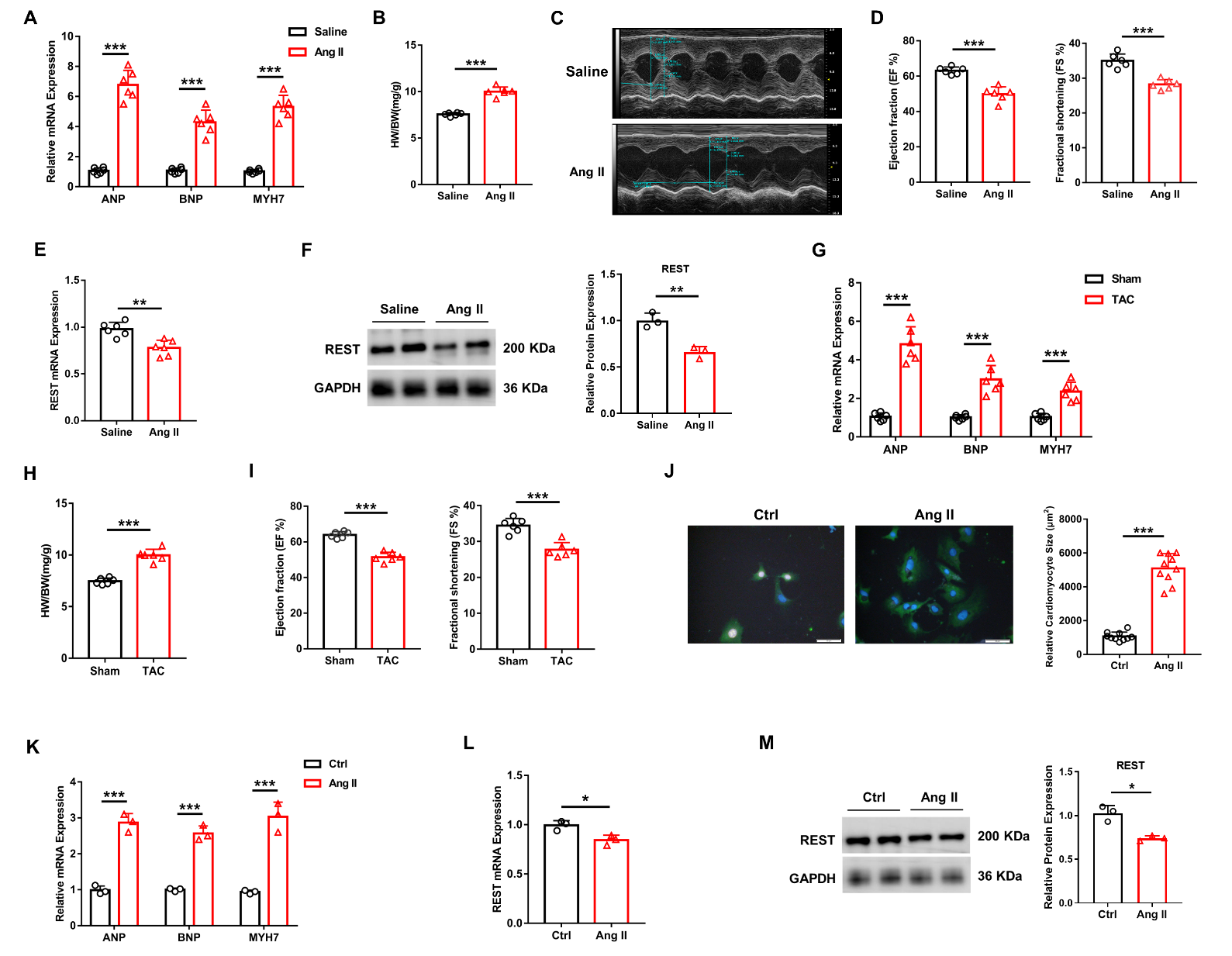
**

**Figure S1. REST expression status in hypertrophic heart and cardiomyocytes.**

(A) Relative mRNA levels of ANP, BNP and MYH7 in Ang II–infused mouse hearts (n = 6). (B) Ratio of heart weight to body weight (HW/BW) in Ang II–infused mouse hearts (n=6). (C, D) Representative M-mode echocardiography of left ventricular chamber, and measurement of ejection fraction (EF%) and fractional shortening (FS%) (n = 6). (E, F) qRT‐PCR and Western blot analysis of REST expression in Ang II–infused mouse hearts (n = 6). (G) Relative mRNA levels of ANP, BNP and MYH7 at week 4 after TAC surgery (n = 6). (H) Ratio of HW/BW with TAC (n=6). (I) Representative M-mode echocardiography measurement of EF% and FS% (n = 6). (J) Representative immunofluorescence images of α-actinin staining of NRCMs infected with anti-TroponinT (green) and DAPI (blue) and treated with AngII (1 μM) or PBS for 48 h. Scale bar, 50 μm. Quantification of myocyte surface area (n=3). (K) Relative mRNA levels of ANP, BNP and MYH7 in NRCMs after Ang II stimulation for 48 hours. (L, M) qRT‐PCR and Western blot results of REST expression in NRCMs after Ang II stimulation for 48 hours. * P < 0.05, ** P < 0.01, *** P < 0.001. All data represent the mean ± standard deviation.


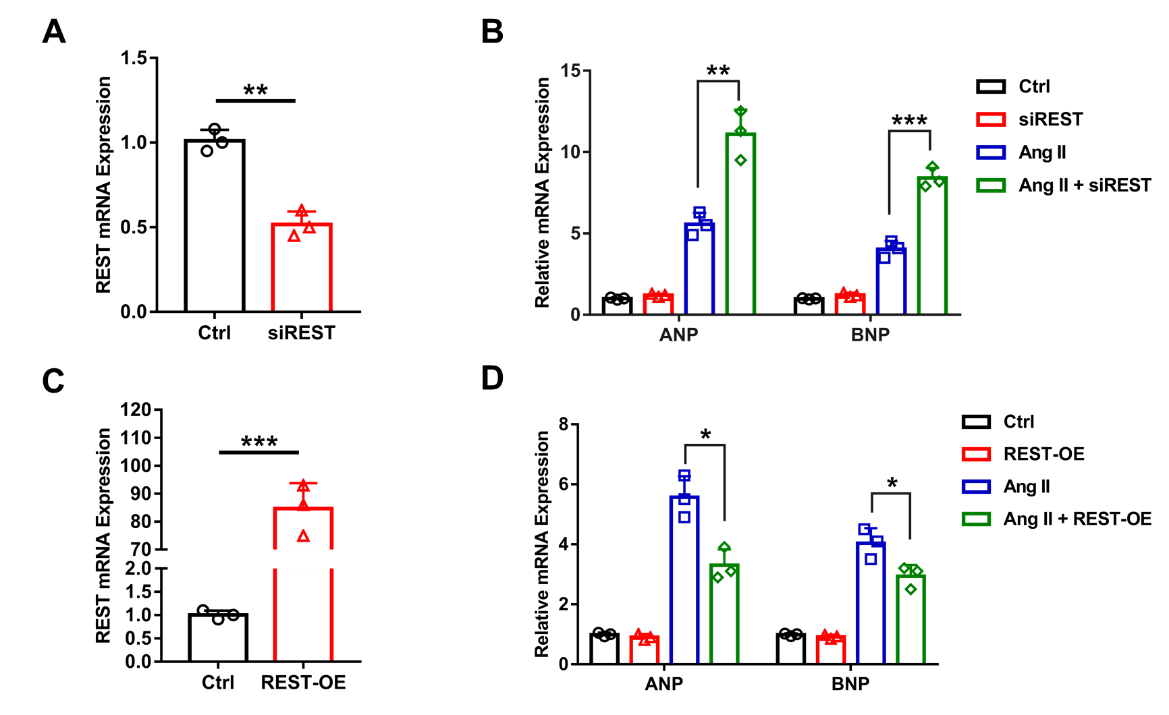


**Figure S2. Effect of REST on cardiac hypertrophy in vitro.**

(A) qPCR results of REST mRNA level in NRCMs infected with siRNA-REST or siRNA-control for 24 hours. (B) The change of ANP and BNP mRNA expression in NRCMs infected with siRNA-REST after Ang II (1 μM) stimulation for 48 hours. (C) REST mRNA level in NRCMs infected with pCMV-Flag-REST or pCMV-Flag-control. (D) The expression levels of ANP and BNP in NRCMs infected with pCMV-Flag-REST after Ang II (1 μM) stimulation for 48 hours. * P < 0.05, ** P < 0.01, *** P < 0.001. All data represent the mean ± standard deviation.


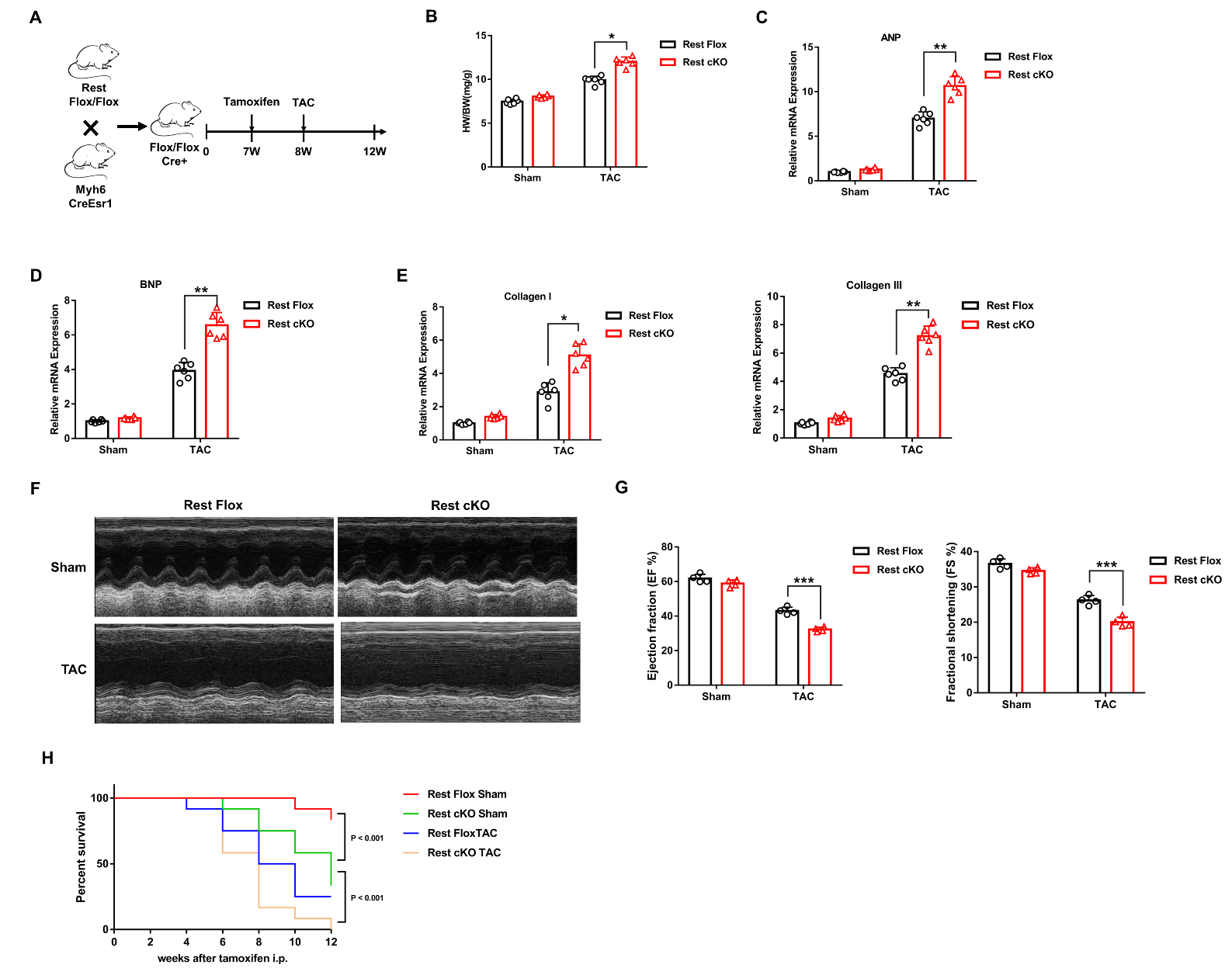


**Figure S3. The effect of REST deletion on cardiac hypertrophy and fibrosis in mouse hearts.**

(A) Schematic diagram of the construction of cardiac hypertrophy model based on cardiac-specific REST knockout (REST f/f: α-MHC-Cre-ER). (B) HW/BW ratios in REST Flox and REST cKO mice at week 4 after sham or TAC surgery (n=6). (C-E) qPCR results of ANP and BNP expression and levels of collagen I and collagen III in same conditions. (F, G) Representative M-mode echocardiography of left ventricular chamber, and measurement of EF% and FS% (n = 4). (H) Kaplan-Meyer survival curves analysis (n=12 for WT; n=12 for REST cKO; n=12 for WT with TAC; n=12 for REST cKO with TAC). * P < 0.05, ** P < 0.01. All data represent the mean ± standard deviation.


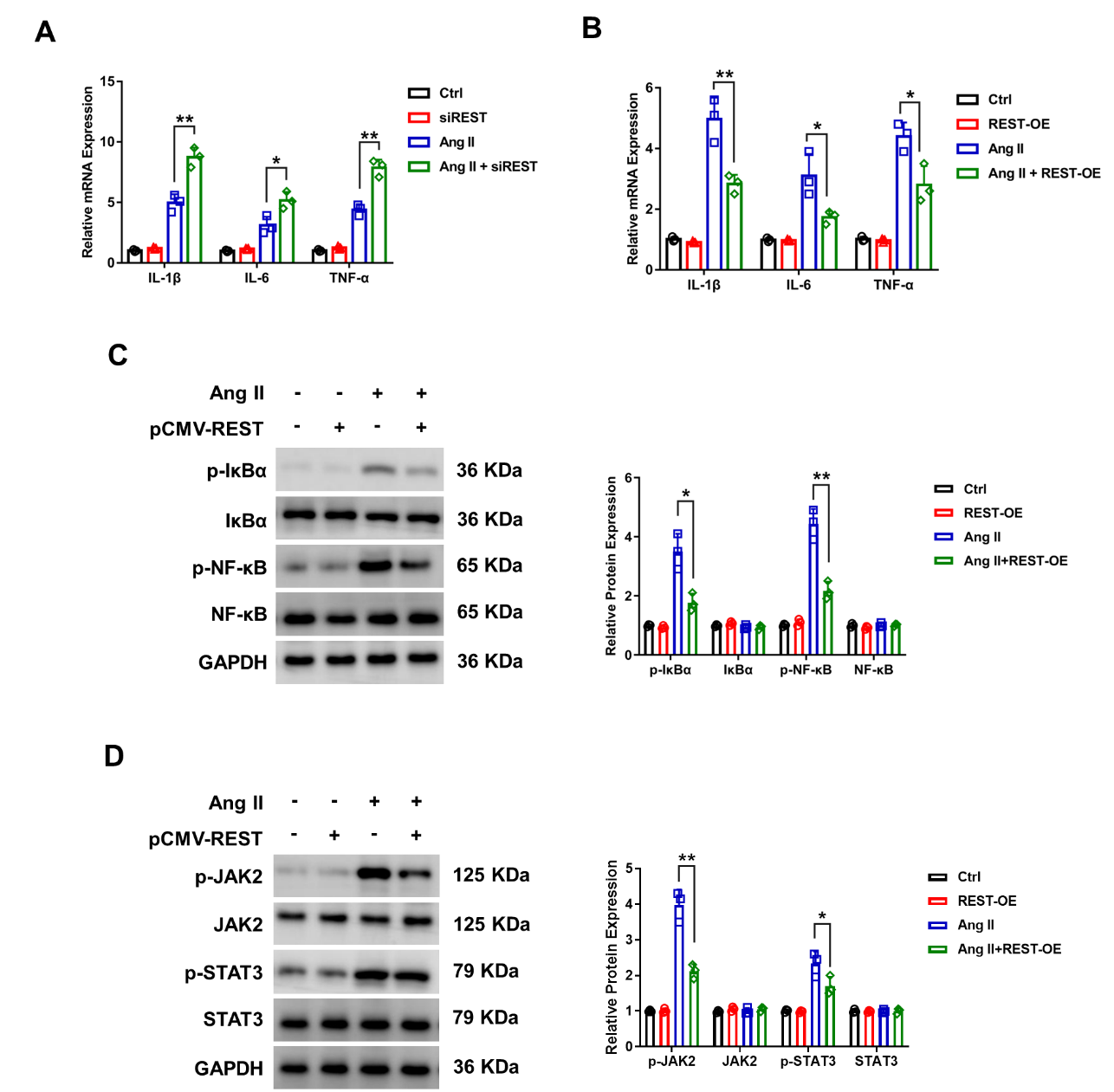


**Figure S4. NF-κB/STAT3 involves in the regulation of REST in cardiac hypertrophy.**

(A) qPCR analysis for inflammatory factors level in NRCMs with inhibition of REST. (B) The changes of inflammatory factors level in Ang II-treated NRCMs with overexpression of REST. (C) The phosphorylation levels of IκBα and NF-κB in Ang II-treated NRCMs with overexpression of REST. (D) The phosphorylation levels of JAK2 and STAT3 in Ang II-treated NRCMs infected with pCMV-Flag-REST. * P < 0.05, ** P < 0.01. All data represent the mean ± standard deviation.


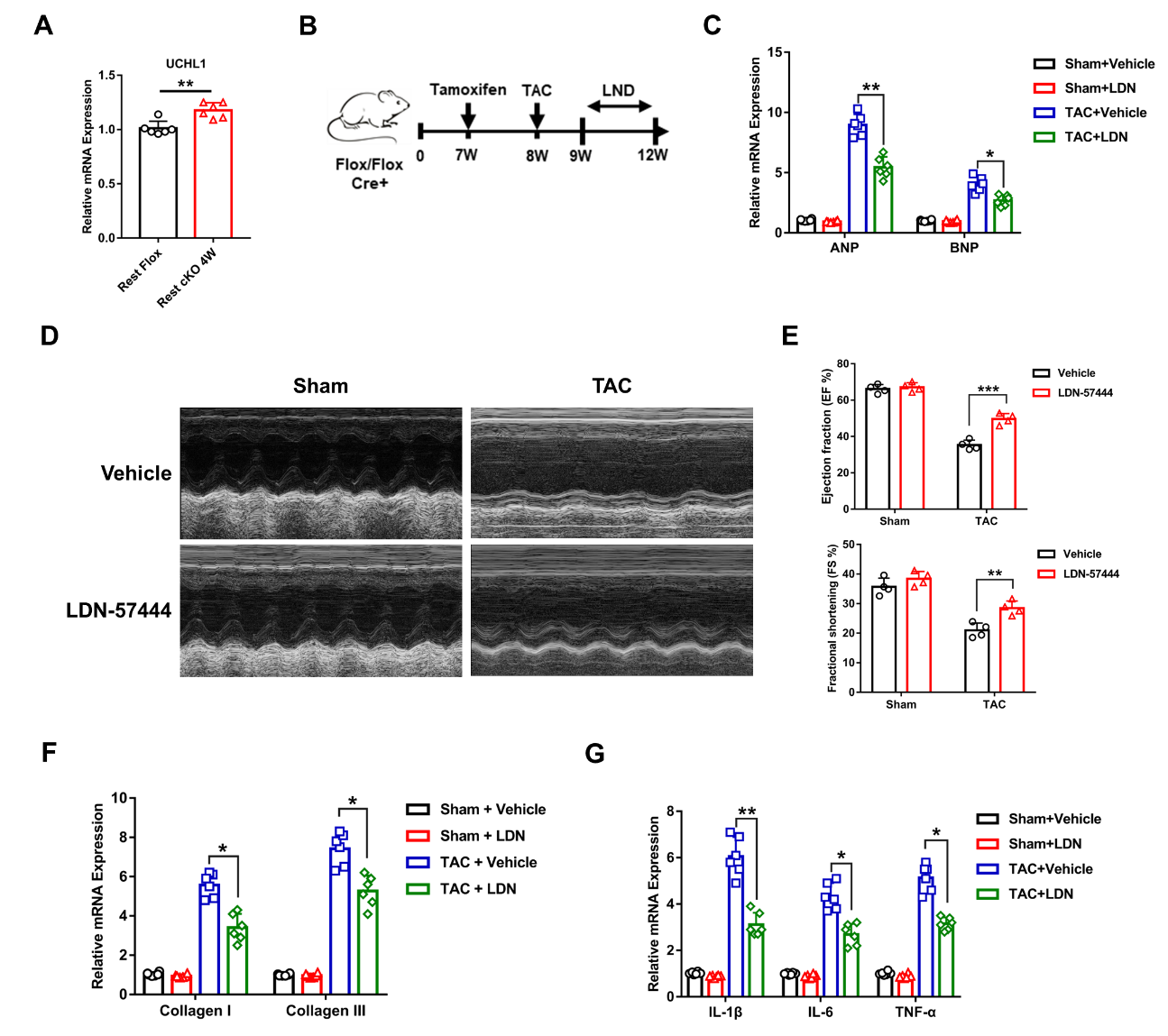


**Figure S5. UCHL1 inhibitor reverses cardiac hypertrophy and inflammation in REST cKO TAC mice.**

(A) Expression levels of UCHL1 in 4-week REST Flox and REST cKO mice (n=6). (B) Schematic diagram of the LDN-57444 administration in REST cKO mice. (C) Levels of ANP and BNP in REST cKO TAC mice with LDN-57444 administration (n=6). (D, E) Representative M-mode echocardiography of left ventricular chamber, and measurement of EF% and FS% (n = 4). (F, G) Levels of collagen I, collagen III and inflammatory factors (IL-1β, IL-6 and TNF-α) in REST cKO TAC mice with LDN-57444 administration (n=6). * P < 0.05, ** P < 0.01. All data represent the mean ± standard deviation.

**
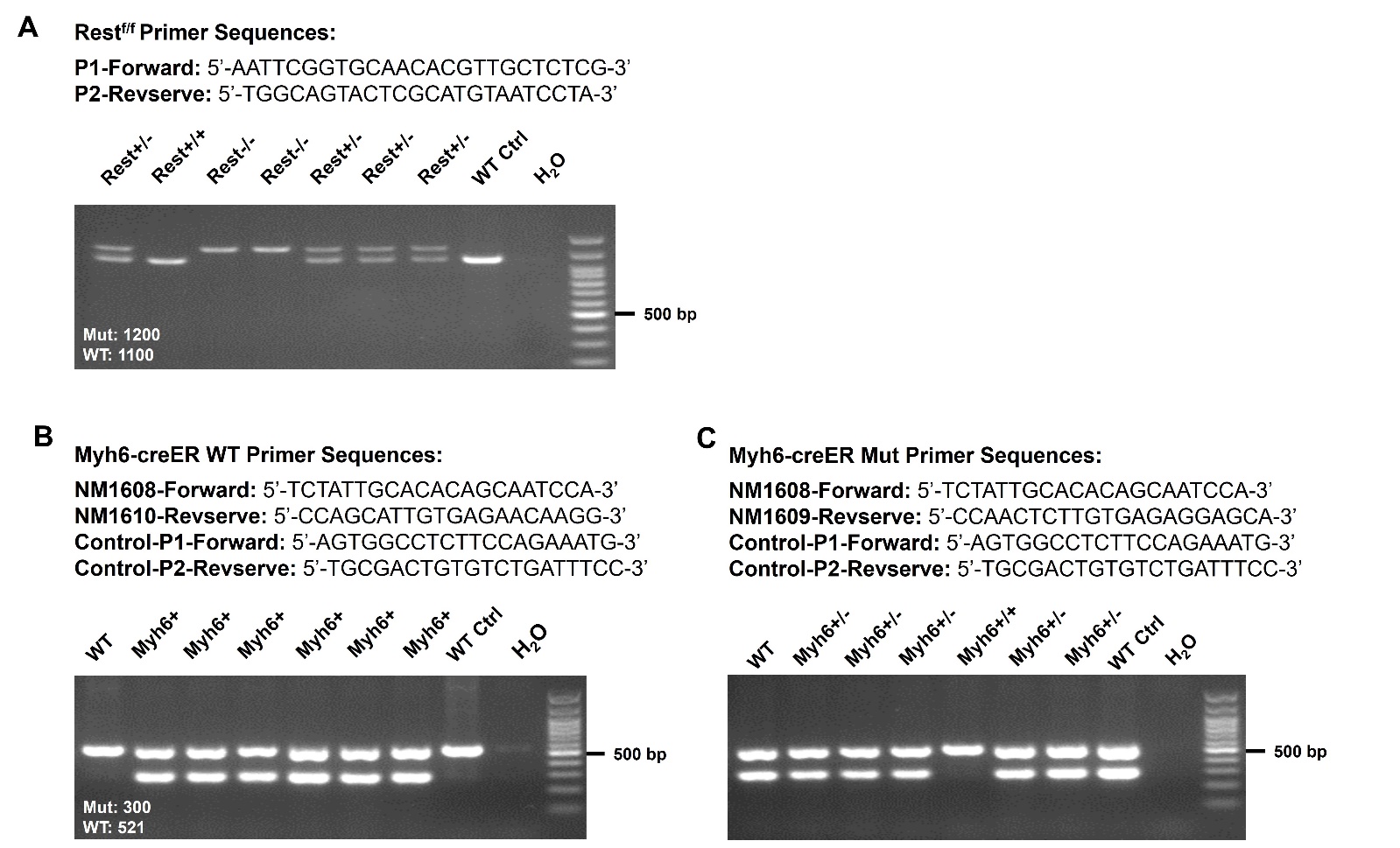
**

**Figure S6. Genotyping analysis of Rest^f/f^ and Myh6-creER mice.**

**Table S1. Primers used for quantitative real-time PCR analysis**

| Gene | Forward Primer | Reverse Primer |
| --- | --- | --- |
| Rat |  |  |
| REST | GCAAACTTGCTCCGAAGTGG | AGATCCTGATGGGGTGACCA |
| ANP | CCCTCCGATAGATCTGCCCT | TTCGGTACCGGAAGCTGTTG |
| BNP | CAATCCACGATGCAGAAGCTG | GGCGCTGTCTTGAGACCTAA |
| MYH7 | TACTTGCTACCCTCAGGTGG | ATGGCTGAGCCTTGGATTCTC |
| IL-1β | TTGAGTCTGCACAGTTCCCC | GTCCTGGGGAAGGCATTAGG |
| IL-6 | CACTTCACAAGTCGGAGGCT | AGCACACTAGGTTTGCCGAG |
| TNF-α | GGCTTTCGGAACTCACTGGA | CCCGTAGGGCGATTACAGTC |
| GAPDH | AAGGTCGGTGTGAACGGATT | CTTTGTCACAAGAGAAGGCAGC |
| Mouse |  |  |
| REST | CGCACAGTTCAGAGGAGTACA | TGCAGGTCGTACATGTCGTT |
| ANP | TCCTAAGCCCTTGTGGTGTG | AAGACCCCACTAGACCACTCA |
| BNP | TTTGGGCTGTAACGCACTGAA | CACTTCAAAGGTGGTCCCAGA |
| IL-1β | TGCCACCTTTTGACAGTGATG | ATGTGCTGCTGCGAGATTTG |
| IL-6 | CCCCAATTTCCAATGCTCTCC | CGCACTAGGTTTGCCGAGTA |
| TNF-α | CCTCACACTCACAAACCACCA | ACAAGGTACAACCCATCGGC |
| Collagen I | AGCACGTCTGGTTTGGAGAG | GACATTAGGCGCAGGAAGGT |
| Collagen III | TGTGGACATTGGCCCTGTTT | TGGTCACTTGCACTGGTTGA |
| UCHL1 | GTTTCGAGAAGAACGAGGCCA | TCACTGGAAAGGGCATTCGC |
| GAPDH | GGGTCCCAGCTTAGGTTCAT | AATCCGTTCACACCGACCTT |

**Table S2. Antibodies used in this study.**

| ANTIBODY | SOURCE | IDENTIFIER |
| --- | --- | --- |
| GAPDH | Proteintech | 60004-1-Ig |
| REST | Proteintech | 22242-1-AP |
| Goat Anti-Mouse IgG Secondary Antibody | Proteintech | SA00001-1 |
| Goat Anti-Rabbit IgG Secondary Antibody | Proteintech | SA00001-2 |
| Donkey Anti-Goat IgG Secondary Antibody | Proteintech | SA00001-3 |
| IKB alpha | Abcam | ab7217 |
| IKB alpha (phospho S36) | Abcam | ab133462 |
| NF-kB p65 (phospho S536) | Abcam | ab86299 |
| NF-κB p65 (D14E12) | Cell Signaling Technology | 8242T |
| Stat3 (124H6) | Cell Signaling Technology | 9139S |
| Phospho-Stat3 (Tyr705) (D3A7) | Cell Signaling Technology | 9145S |
| Jak2 (D2E12) | Cell Signaling Technology | 3230S |
| Phospho-Jak2 (Tyr1007/1008) | Cell Signaling Technology | 3771S |
